# Reconstruction of the Fatty Acid Biosynthetic Pathway of* Exiguobacterium antarcticum* B7 Based on Genomic and Bibliomic Data

**DOI:** 10.1155/2016/7863706

**Published:** 2016-08-09

**Authors:** Regiane Kawasaki, Rafael A. Baraúna, Artur Silva, Marta S. P. Carepo, Rui Oliveira, Rodolfo Marques, Rommel T. J. Ramos, Maria P. C. Schneider

**Affiliations:** ^1^Genomics and Systems Biology Center, Institute of Biological Sciences, Federal University of Pará, 66075-110 Belém, PA, Brazil; ^2^Rede de Química e Tecnologia/Centro de Química Fina e Biológica, Chemistry Department, Universidade Nova de Lisboa, 2829-516 Costa da Caparica, Portugal

## Abstract

*Exiguobacterium antarcticum* B7 is extremophile Gram-positive bacteria able to survive in cold environments. A key factor to understanding cold adaptation processes is related to the modification of fatty acids composing the cell membranes of psychrotrophic bacteria. In our study we show the* in silico* reconstruction of the fatty acid biosynthesis pathway of* E. antarcticum* B7. To build the stoichiometric model, a semiautomatic procedure was applied, which integrates genome information using KEGG and RAST/SEED. Constraint-based methods, namely, Flux Balance Analysis (FBA) and elementary modes (EM), were applied. FBA was implemented in the sense of hexadecenoic acid production maximization. To evaluate the influence of the gene expression in the fluxome analysis, FBA was also calculated using the log_2_⁡FC values obtained in the transcriptome analysis at 0°C and 37°C. The fatty acid biosynthesis pathway showed a total of 13 elementary flux modes, four of which showed routes for the production of hexadecenoic acid. The reconstructed pathway demonstrated the capacity of* E. antarcticum* B7 to* de novo* produce fatty acid molecules. Under the influence of the transcriptome, the fluxome was altered, promoting the production of short-chain fatty acids. The calculated models contribute to better understanding of the bacterial adaptation at cold environments.

## 1. Introduction

Bacteria are increasingly used in industrial processes to produce chemicals, foods, and drugs, among other products [1]. The main biochemical pathways of bacteria may be manipulated and optimized to more efficiently produce compounds of industrial interest in various areas; for example, metabolic pathways of* Corynebacterium glutamicum* are rationally engineered to produce L-amino acids on an industrial scale [2]. To accomplish this task, specific tools are used such as FMM (from metabolite to metabolite) [3] Cytoscape [4], CellDesigner [5], SBW (Systems Biology Workbench) [6], COPASI (COmplex PAthway SImulator) [7], and COBRA (COnstraints Based Reconstruction and Analysis) toolbox [8].

The genomes of several bacterial strains have been sequenced and annotated and have been used in combination with biochemical and physiological data to reconstruct metabolic networks at a genome-scale [9]. Recently, genomic models were reconstructed for some bacterial species aiming to increase the amount and quality of data that has been annotated in either the literature or databases [10–12]. The draft network generated from the annotated genome still requires significant manual curation for a comprehensive and accurate metabolic representation of the organism [13].

The need to develop automatic or at least semiautomatic methods to reconstruct metabolic networks from genome annotation is increasing because the number of complete genome sequences available is growing fast. Recent studies [13, 14] have highlighted the problems associated with genome annotations and databases, which perform automatic reconstructions and, thus, require manual assessment. The currently available 96-step protocol, proposed by Thiele and Palsson [15], is a well-established process for the assembly, curation, and validation of metabolic reconstruction. This protocol is combined with computational tools, including the visualization and numerical calculation software package, MATLAB® (MathWorks, USA).

Constraint-based modeling is frequently used as final validation step of the reconstructed network. This step is extremely useful for simulating the phenotypic behavior under different physiological environments [16–18] thereby allowing assessing if the reconstructed network represents well the* in vivo* cellular system. Microbial adaptation to cold environments is one of the applications of these methods.

Psychrotrophic microorganisms have an optimal growth temperature higher than 15°C but are also able to grow and adapt to extremely cold environments, with temperatures of approximately 0°C [19]. Thus, the unique physicochemical characteristics of their habitat and the biological apparatus developed by these microorganisms to survive under these conditions render these organisms valuable sources of biotechnological processes. The cellular response to cold by psychrotrophic bacteria may be studied from a general standpoint with the advent of omics methods. Recently, the B7 strain of* Exiguobacterium antarcticum* was isolated from Ginger Lake sediments located in the Antarctic Peninsula Region (69°30′S, 65°W). This lake was formed due to the warming in the region, which led to partial melting of ice caps [20]. The genome of this strain was sequenced [21], and its response to cold was evaluated through differential expression of its genome at 37°C and 0°C using omics methods [22]. One of the mechanisms of cold adaptation of all psychrophilic or psychrotrophic organisms is the change in the chemical structures of the membrane phospholipids. The fatty acid chains become shorter and unsaturated at low temperatures. Accordingly, the fluidity of the membrane is kept intact [19, 23].

Bacterial* de novo *synthesis of fatty acids is regulated by the protein FapR [24], which is responsible for activating/disabling a regulon consisting of four operons in* E. antarcticum *B7. In cold, two of these operons are repressed (*fabH1-fabF* and* fabI*), and the expression levels of the other two remain unaltered [22]. The chemical components, which are included in this regulon, must be reconstructed and evaluated and then associated with their respective genetic elements to further understand this metabolic pathway and to reach more complete conclusions about its importance for adaptation to cold [23, 24]. Bioinformatics methods are used for* in silico* reconstruction of metabolic pathways [25].

In this work we present the* in silico* reconstruction of the fatty acid biosynthesis pathway of the* Exiguobacterium antarcticum* B7, based on linear programming (FBA) and convex cone method (elementary modes). The influence of transcriptome in FBA calculation was also evaluated.

## 2. Materials and Methods

### 2.1. Data Collection

The genomic data of* E. antarcticum* B7 in the formats  .gbk and  .fasta were collected from NCBI under accession number NC_018665. The metabolic pathway of the fatty acid biosynthesis was initially evaluated in the KEGG (Kyoto Encyclopedia of Genes and Genomes) database [26]. When necessary, the visualization of the genome of the bacterium was performed using Artemis software [27].

### 2.2. Preliminary Reconstruction

This step was performed following two methods: one semiautomatic and the other automatic. The tools within the KEGG databases were used essentially in the semiautomatic method. The.fasta file with the* E. antarcticum* B7 genome was submitted to the online tool KAAS (KEGG Automatic Annotation Server) [28], available at http://www.genome.jp/kaas-bin/kaas_main. The parameters chosen to run this software were as follows: (a) bidirectional Best Hit (BBH) Method, recommended for complete genomes, performs the search for orthologous genes between a specific group of organisms, and (b) prokaryote, the set of genes chosen, should be representative of the target organism, in this case, the bacterium* E. antarcticum* B7. Following the processing, a text file was generated (query.ko). Each line of this file is formed by two parameters: the first consists of the sequence identified (gene), and the second, when present, consists of the KO assignment, termed *K* number. This value indicates orthologous groups encoding the same enzymatic activity. Afterwards, the file generated is passed through a filter, an auxiliary computer software program (script) Python developed for the present study, which only selects *K* numbers and individually and increasingly commands per line into a new file (new_query.ko). This file was used as entry in the option User Data Mapping of the Pathway Mapping tool of the KEGG.

The automatic method essentially consisted of submitting the  .fasta file of the* E. antarcticum* B7 genome to the online tool RAST (Rapid Annotation using Subsystems Technology) [29], available at http://rast.nmpdr.org/, to generate the drafts of the metabolic network and of the fatty acid biosynthesis pathway of the target microorganism. The final draft of this step was generated from the combination of the resulting pathways of the semiautomatic and automatic models. The common pathways were maintained, while surplus compounds, enzymes, and reactions, that is, present in some, but absent in others, were not directly excluded but were instead reserved for the curated step.

### 2.3. Manual Curation

The following steps were completed in this manual curation stage, following the protocol explained above. (i) Draft refinement: this phase began with the analysis of enzymes and reactions, components of the fatty acid biosynthesis pathway, by reading books and articles specifically on the subject. The objective was to diagnose the absence or presence of more than one element of the study pathway. The online databases KEGG, ENZYME [30], and SEED [31] were consulted to ratify the enzymes and the structures of the reactions.

(ii) Assessment of the stoichiometry and reversibility of the reactions: in this step, all model reactions were assessed and stoichiometrically corrected, if necessary. The biochemical data on the organism are very important to determine the reversibility of the reaction. For this purpose, the databases (KEGG, SEED, and ENZYME) and the tool eQuilibrator [32] were used to analyze the reversibility of the reactions. Thus, the thermodynamic constraints were respected.

(iii) Addition of gene data and reaction location: the Artemis tool was used to identify the genes of the reactions (enzymes) from their locations in the genome assessed using the draft generated.

(iv) Assessment of Gene-Protein-Reaction (GPR) associations: in this step, the function of each gene is indicated. GPR associations were identified using databases of the organism and specific literature.

(v) Definition of constraints: some constraints were defined in the model in this manual curation step, including stoichiometric and thermodynamic constraints (through the reversibility and irreversibility of fluxes).

### 2.4. Metabolic Model Design

The metabolic model designed and refined following the manual curation step was converted into a mathematical representation, termed a stoichiometric matrix, which encouraged the development of a wide variety of computational tools to analyze network properties.

The constraints of capacity, which are the upper and lower limits defining the maximum and minimum fluxes allowed for the reactions, were added in this step. The inputs of the stoichiometric matrix are the coefficients of the metabolites in the reactions with negative values for consumed metabolites (substrates) and positive values when the metabolites are produced or secreted (products) (Additional File 1 see Supplementary Material available online at http://dx.doi.org/10.1155/2016/7863706).

### 2.5. Metabolic Pathway Validation

The computational model sought to examine the metabolic capabilities and to evaluate the system properties they may perform under the constraints imposed on the cell. Thus, the final step in the reconstruction process consisted of assessing, evaluating, and validating the fatty acid biosynthesis pathway of* E. antarcticum* B7. The validation of that metabolic model was performed using simulation and flux analysis. The fatty acid biosynthesis pathway is well described in the literature because it is a highly conserved process among organisms, which facilitated its complete definition. Thus, most gaps had already been filled during the manual curation process.

## 3. Results and Discussion

The expectation to understand the relationship between the genome and the physiology of a particular organism was a key incentive for reconstructing metabolic networks. Protocol adaptations using semiautomatic and automatic methods are necessary to reconstruct the metabolic networks of organisms with few reported data on their metabolic capabilities, including* E. antarcticum* B7.

### 3.1. Pathway Reconstruction Using the Semiautomatic Method

**Figure 1:**
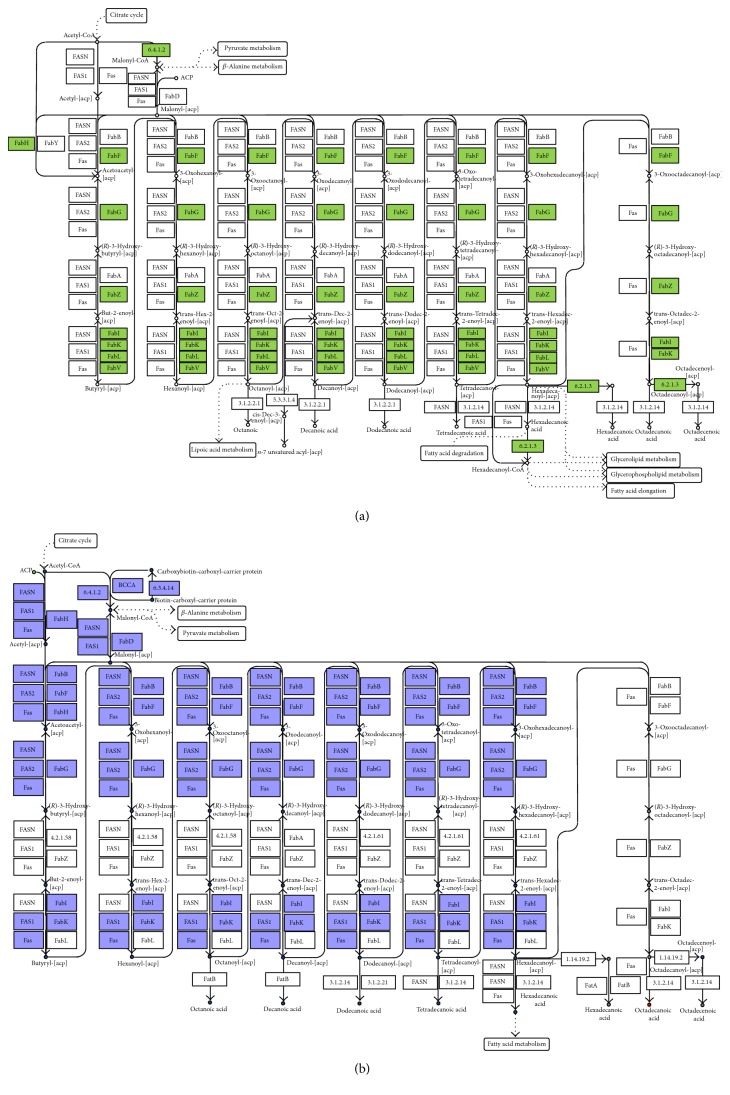
Drafts of the fatty acid biosynthesis pathway of* E. antarcticum* B7 bacteria. The drafts were designed using the following methods: (a) semiautomatic method, generated by the KEGG database, and (b) automatic method, generated by online tool RAST. Colored boxes indicate the possibility of the presence of enzymes in the pathway.

The draft of the metabolic network of* E. antarcticum* B7 was retrieved from the KEGG database [26]. The KEGG Metabolic Pathway tool was used to highlight the fatty acid biosynthesis pathway from the resulting draft of the metabolic network. The genes annotated and identified using KEGG and their respective enzymes are shown in Figure 1(a) in green, and the others are listed in white boxes.  Table 1 shows the genes, locus tags, and enzymes identified using the KEGG Metabolic Pathway tool. A total of 11* locus tags* associated with their respective genes and Enzyme Commission (EC) numbers were identified; only the locus tag Eab7_2235 has no added gene associated with it.

### 3.2. Pathway Reconstruction Using the Automatic Method

The RAST/SEED tool does not provide graphic display of the metabolic map draft as KEGG; for this purpose, it uses a standard table to list the 247 metabolic pathways that compose the network, regardless of whether they were identified in the genome of the microorganism. RAST identified 25 reactions, 40 compounds, and 20 EC numbers in the fatty acid biosynthesis pathway of* E. antarcticum* B7.  Table 1 outlines the genes, locus tags, and enzymes identified at this step. Annotated genes identified by RAST and their respective enzymes are colored in purple, and the others are listed in white boxes (Figure 1(b)).

The analysis of both fatty acid biosynthetic pathway drafts shows that the draft generated using KEGG apparently has the most complete flux, except for enzyme 6.3.4.14, which is exclusively present in the draft resulting from the RAST tool. The draft generated using RAST has a gap in which the enzymes FabA and FabB are not included in the pathway elongation process. The flux for the production of hexadecenoic acid is also absent from the pathway generated using RAST.

Artemis software was used to confirm the presence of all genes selected through the automatic and semiautomatic methods. The genes accABCD, fabD, fabH1, fabF, fabG, fabZ, and fabI and the locus tag Eab7_2235 were described in the genome of* E. antarcticum* B7, except fabK gene, which was detected only by the automatic method.

The KGML file produced by KEGG was submitted to the software KEGGtranslator [33] to be converted into a SBML (System Biology Markup Language) file [34]. This file was converted into an Excel spreadsheet using MATLAB functions. The files in SBML format and the Excel spreadsheets are the most used formats in metabolic reconstructions. The reactions and metabolites of the preliminary network generated using KEGG could be visualized in the spreadsheet.

The data generated using the RAST/SEED tool were analyzed and added to the first step of the process, supplementing the data collected using KEGG. The files generated with both platforms were used to manage the manual curation data.

The larger number of genes identified using KEGG (12) compared to those found using RAST/SEED (9) may be explained because the former uses orthology (KEGG Orthology (KO)) through protein homology to identify the so-called metabolite genes [35] in a genome, which facilitates finding gene-protein-reaction (GPR) associations.

### 3.3. Manual Curation of the Metabolic Pathway of* De Novo* Fatty Acid Synthesis

The reactions of the fatty acid synthesis pathway were annotated and refined. The metabolites were organized into two compartments (cytoplasm and extracellular compartment) based on the location of the enzymes associated with each pathway. The cofactors and the reversibility of the reactions were compiled from the data published in the literature and online tools (ENZYME and BRENDA). The EC number was noted, and the genes were identified. A summary of those results is shown in Table 2. Thermodynamic analysis of the reactions revealed that malonyl-CoA synthesis from acetyl-CoA (AcCoA) is an irreversible process; similar to the process regarding the* fabF* gene, the Eab7_2235 locus tag, and the extracellular metabolites the other processes are reversible.

It is very important to assess the quality of the annotated genome submitted to the online tools during curation. The literature categorically states that the quality of the reconstructed network directly depends on the annotated genome of the organism. The rule is to use the latest updated version of the annotated genome [36–38].

### 3.4. Curated Metabolic Network

The previous step added constraints regarding the stoichiometry (chemical balance), thermodynamics (reversibility of reactions), and physiology (cofactors used) of the study model. The result was a system of equations that describes the cell metabolism according to the metabolites of interest. The mathematical representation of this model essentially consisted of describing the performance of the fatty acid biosynthesis pathway using a stoichiometric matrix. This data structure consists of 54 metabolites and 59 reactions, resulting in a 54 × 59 stoichiometric matrix (Additional File 1). Additional File 2 shows the list of biochemical reactions identified in the curated model.

**Figure 2:**
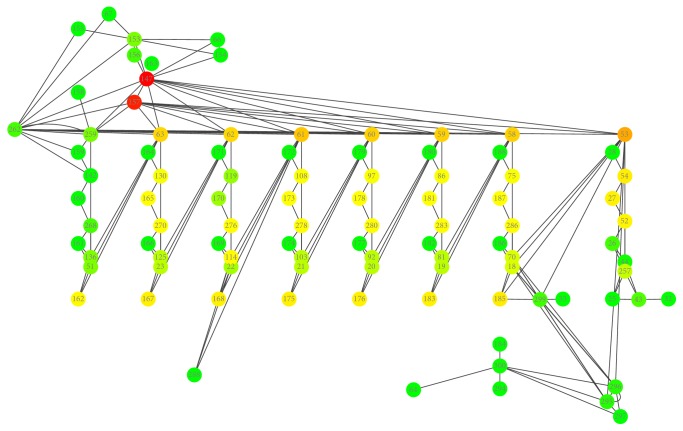
Layout of the fatty acid biosynthesis pathway generated using Cytoscape. Green vertices: fewer connections. Yellow vertices: regular number of connections. Red vertices: large number of connections. This network is a free model scale.

The reconstructed pathway model was converted into SBML in the used MATLAB toolbox. The SBML file was validated using the tool SBML validator and was then submitted to the tool Cytoscape, which generated the network of Figure 2. The gene-protein-reaction (GPR) representation therein describes the degree of connectivity of each enzyme in the pathway. Vertices with few connections are in green, the vertices with regular numbers of connections are in yellow, and the vertices with large numbers of connections are in red. The network connectivity obeys a scale-free model [39].

### 3.5. Flux Balance Analysis (FBA)

The FBA was coded in MATLAB implementing a constrained linear program using the GLPK (GNU Linear Programming Kit) linear optimization library [8]. All fluxes were calculated in percentage of the input flux of AcCoA (reaction 39), which was fixed to 100. Hexadecenoic acid is the key metabolic product; thus the respective flux (reaction 41) was set as the maximization target for FBA. To improve convergence, upper and lower bounds were [0,100] for irreversible reactions and [−100,100] for reversible reaction. The final optimized fluxes are shown in Additional File 3. The target maximum, reaction R41 in Additional File 3, was 7.69, which may be read as 7.69 moles of hexadecenoic acid produced for every 100 moles of AcCoA consumed per unit time per unit cell mass.

**Figure 3:**
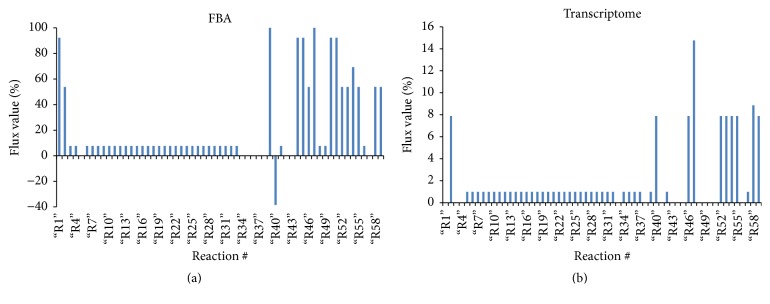
Flux graph generated using the software MATLAB, which uses methods based on FBA constraints. The vertical axis represents the fluxes calculated from the input of 100 moles of acetyl-CoA (AcCoA). The horizontal axis represents the reactions participating in the fatty acid biosynthesis pathway. The blue bars determine the output percentage for each pathway reaction. (a) FBA for maximizing the production of hexadecenoic acid. (b) FBA calculated with the log_2_⁡FC values obtained from the transcriptome.

Figure 3(a) shows the generated flux plot, which shows the variation occurring between the response fluxes, with the majority, approximately 37, showing positive values smaller than 20, while 15 are above that range.

### 3.6. Influence of Transcriptome in FBA Calculation

Log base 2-fold change values (log_2_⁡FC) obtained* in vitro* by Dall'Agnol and colleagues [22] were used to evaluate the influence of differential expression in the FBA calculation. These values were obtained by comparison of RPKM (reads per kilobase per million reads sequenced) generated in the transcriptome of the bacterium at 0°C and 37°C. The log_2_⁡FC of genes that composes the fatty acid biosynthesis pathway is shown in Table 2.

As presented by Dall'Agnol and colleagues [22], the aerobic energetic metabolism of* E. antarcticum* B7 at 0°C is repressed, and a fraction of the acetyl-CoA is probably used as a substrate to synthesize short-chain fatty acids in cold. The synthesis begins with the conversion of acetyl-CoA to malonyl-CoA catalyzed by the multimeric enzyme encoded by the genes* accABCD*. In FBA analysis, only half of the input of acetyl-CoA (100 mM) is converted to malonyl-CoA, which binds to the acyl carrier protein (ACP) at 0°C (Figure 3(b)). As stated earlier, the other half of acetyl-CoA is probably used for energy generation.

The remaining route is cyclical, being the reactions catalyzed by enzymes encoded by* fabF*,* fabG*,* fabZ*, and* fabI* genes. At each round, two carbons are added to the growing chain of fatty acid. In these reactions, the flow of metabolites remains unchanged until the fatty acid molecule reaches a size of eight carbons (octadecanoyl-ACP in reaction 38) where the percentage of the flow amount decreases (Figure 3(b)). These results are consistent with the previously published data which affirm that bacteria decrease their fatty acid chains to survive in cold. These short fatty acid molecules will be converted into membrane phospholipids in order to maintain the fluidity of this biological barrier.

### 3.7. Elementary Flux Modes

The calculation of elementary modes was performed in MATLAB using the Metatool toolbox [40] (*modo_elementar.m* code in the Supplemental Information). A total of 13 elementary flux modes were found for the fatty acid biosynthesis pathway of* E. antarcticum* B7 (Additional File 4). Of these, only 4 elementary modes (2, 5, 8, and 11) have a positive nonzero coefficient for reaction 41, which indicates that the target product hexadecenoic acid may only be generated by one of these four elementary modes. The routes identified in EM2 and EM5 begin at the second reaction (R2) which is catalyzed by the enzyme FabD. The value of R2 for both elementary modes indicates a considerable production of malonyl-CoA. The remaining reactions of EM2 and EM5 are presented in Additional File 4. In EM8 and EM11, the routes begin from R1. The value 1 for this reaction indicates a lower activity, reflecting in a lower production of hexadecenoic acid. Regarding reaction 41, the values of elementary modes 2 and 5, in this case 1, are higher than the values of 8 and 11 (0.142857 each), indicating that both elementary modes 2 and 5 produce 1 mol hexadecenoic acid when they are active, while elementary modes 8 and 11 produce 0.142857 moles.

## 4. Conclusions

The first metabolic pathway of* E. antarcticum* B7, reconstructed following the steps defined in this work, suggests that the protocol used is a suitable tool for further metabolic reconstruction studies. Almost all the first steps of the process were automated; however, manual curation was, as usual, laborious because it required an intensive search for available data.

The metabolic pathway of fatty acid biosynthesis was representative and consistent under the limits and boundary conditions set. The FBA and elementary mode methods were used to examine the hexadecenoic acid production potential of the reconstructed pathway. The application of constraint-based modeling revealed being very useful to assess network operation plasticity, even if the intracellular kinetics are unknown. The* in silico* analysis performed using FBA enabled a quantitative assessment of hexadecenoic acid production potential.

Finally, a key issue involves deciding when to stop the process and to consider the reconstruction finalized, at least temporarily. This decision is usually based on the reconstruction purpose. The most complete metabolic model currently available is the* E. coli* model, which has been researched and refined for over 10 years [41–45]. Other studies constantly updating their models are* Homo sapiens*, with three reconstructions [46–48], and* S. cerevisiae*, with more than a dozen reconstructions, including two in 2013 [49, 50]. The protocol reported in the present study may be used to compile several data pieces available in the literature aimed at proposing possible metabolic pathways, thereby enabling deeper research of the metabolism under study.

## Supplementary Material

The supplementary material contains: a stoichiometric matrix generated after draft analisys (File 1), the biochemical reactions identified in the draft model and included in the stoichiometric matrix (File 2), and the calculated FBA rates (File 3). All these documents are in .xls format.

## Figures and Tables

**Table 1:** Genes, locus tags, and EC numbers identified in the draft of the fatty acid biosynthesis pathway of *E. antarcticum* B7. The features displayed were generated using the methods: semiautomatic and automatic.

Semiautomatic method			Automatic method		
Gene	*Locus tag*	EC number	Gene	*Locus tag*	EC number
accA	Eab7_2059	6.4.1.2	accA	Eab7_2059	6.4.1.2
accB	Eab7_0870	6.4.1.2	accB	Eab7_0870	6.4.1.14
accC	Eab7_0871	6.4.1.2	accC	Eab7_0871	6.4.1.2
accD	Eab7_2060	6.4.1.2	accD	Eab7_2060	6.4.1.14
fabD	Eab7_1760	2.3.1.39	fabD	Eab7_1760	2.3.1.39
fabH1	Eab7_1911	2.3.1.180	fabH1	Eab7_1911	2.3.1.180
fabF	Eab7_1910	2.3.1.179	fabF	Eab7_1910	2.3.1.179
fabG	Eab7_1795	1.1.1.100	fabG	Eab7_1795	1.1.1.100
fabZ	Eab7_2463	4.2.1.59	fabI	Eab7_1885	1.3.1.10
fabI	Eab7_1885	1.3.1.10			
fabK	Eab7_0377	1.3.1.9			
—	Eab7_2235	1.14.19.2			

**Table 2:** Relationships between components of the *E. antarcticum* B7 fatty acid biosynthesis pathway following curation. The signals ⇒ and ⇔ indicate irreversible and reversible reactions, respectively.

Gene	Locus tag	EC number	Enzyme	Reaction	Fold change (log_2_⁡FC)
accA	Eab7_2059	6.4.1.2	Acetyl-CoA carboxylase carboxyl transferase alpha subunit	ATP + acetyl-CoA + HCO_3_^−^⇒ ADP + orthophosphate + malonyl-CoA	0.4562
accB	Eab7_0870	6.4.1.2	Acetyl-CoA carboxylase biotin-carboxyl carrier protein	ATP + acetyl-CoA + HCO_3_^−^⇒ ADP + orthophosphate + malonyl-CoA	−0.05773
accC	Eab7_0871	6.4.1.2 6.4.1.14	Acetyl-CoA carboxylase, biotin carboxylase subunit	ATP + acetyl-CoA + HCO_3_^−^⇒ ADP + orthophosphate + malonyl-CoA	−0.5623
accD	Eab7_2060	6.4.1.2	Acetyl-CoA carboxylase carboxyl transferase beta subunit	ATP + acetyl-CoA + HCO_3_^−^⇒ ADP + orthophosphate + malonyl-CoA	−0.2811
fabD	Eab7_1760	2.3.1.39	ACP S-malonyl transferase	Malonyl-CoA + ACP ⇔ CoA + malonyl-(acp)	0.9448
fabH1	Eab7_1911	2.3.1.180	3-Oxoacyl-ACP synthase III	Acetyl-CoA + malonyl-(acp) ⇔ acetoacetyl-(acp) + CoA + CO_2_	0.8512
fabF	Eab7_1910	2.3.1.179	3-Oxoacyl-ACP synthase II	Acetyl-(acp) + malonyl-(acp) ⇒ acetoacetyl-(acp) + CO_2_ + ACP	0.6942
fabG	Eab7_1895	1.1.1.100	3-Oxoacyl-ACP reductase	Acetoacetyl-(acp) + NADPH + H^+^ ⇔ (R)-3-hydroxybutanoyl-(acp) + NADP^+^	0.8523
fabZ	Eab7_2463	4.2.1.59	3-Hydroxyacyl-ACP dehydratase	(R)-3-hydroxybutanoyl-(acp) ⇔ but-2-enoyl-(acp) + H_2_O	0.12902
fabI	Eab7_1885	1.3.1.9 1.3.1.10	Enoyl-ACP reductase I	But-2-enoyl-(acp) + NADH + H^+^ ⇔ butyryl-(acp) + NAD^+^	0.2969
—	Eab7_2235	1.14.19.2	Acyl-ACP desaturase	Hexadecanoyl-(acp) + acceptor_reduced + O_2_ ⇒ hexadecenoyl-(acp) + acceptor + 2H_2_O	1.3768
